# Surgical Cases

**Published:** 1857-08

**Authors:** J. C. Hughes

**Affiliations:** Prof. of Surgery in Med. Dept. of Iowa State University


### Surgical Cases, (Continued from page 24.)

REPORTED BY J. C. HUGHES, M. D.

Prof. of Surgery in Med. Dept. of Iowa State University.

### *Caries of Cranial Bones.*

CASE 2nd.—Mr. B——, a gentleman aged 45, formerly a merchant of Detroit, applied to me in the latter part of June, 1856, for advice and treatment.

He had been suffering for some two years with what had been pronounced disease of the scalp, and had been treated accordingly, but without any beneficial results.

Upon inquiry into the early history of his case I could find no immediate exciting cause. Some five or six years prior to the presence of these symptoms he had received a fall which had produced fissure of the cranium, near the site of disease, but no symptoms followed which led him to believe that this had anything to do with the present condition of the parts; nor was there present any scrofula, syphalitic or mercurial cachexy, his only vice being that of intemperance.

The location of the disease was upon the upper and anterior portion of the cranium, occupying in the soft parts a space not over an inch in extent, near the junction of the sagital with the coronal suture. The wound presented an unhealthy appearance, and when granulations were present they were of a weak character. The discharge was small, consisting of unhealthy pus, thin and fœtid. When the probe was introduced, I discovered the surface of the bones at the point of opening to be irregular, and was able to pass the instrument, for an inch or more, in every direction; at almost every point I found that rough and diseased condition of bone which is present in caries.

There was, at some points, a softened and spongy feeling to such an extent as to deter me from the application of much force, fearing entire perforation of the cranium, and consequent injury to the brain itself.

Satisfied that I had a case of caries, and one which demanded an operation as the only hope, I placed my patient under constitutional treatment preparatory to the operation, and in some two weeks proceeded to operate.

I made a crucial incision to the extent of some three inches in each direction, then separating the scalp, which I found very loosely attached, the extent of the disease was more fully shown. At some points the softening extended partially through the internal table, the external having been entirely destroyed. With a small chisel and mallet I removed the entire external table of a portion of the frontal and parietal bones, for a space of two and a half by three inches, and by gouge removed the diseased portions of the internal table. Afterwards the wound was closed and the simple water dressings applied. With a supporting constitutional treatment, and a continuance of the local dressings, the patient in a few weeks was entirely restored to health, and at this date, more than one year after the operation, the parts are firm and healthy over the whole extent of the diseased part. But in stopping here, I should not be doing justice to the readers of our Journal, and should be taking that course too frequently adopted by those reporting cases—*never failing to cure.*

Within the last two weeks this same patient presented himself, stating that a small and painful tumor, had presented itself upon the back part of the head. I examined it and found its location to be about the line of the lambdoidal suture, between the occipital and left parietal bones; I found distinct fluctuation present and at once punctured it, when an unhealthy, purulent matter escaped. Upon the introduction of the probe I was not able to detect any disease of the bone, but a softened and spongy condition of the soft parts. There were also discovered, upon examination, two other small swellings upon the after part of the cranium, exhibiting signs of incipient suppuration, which I did not open. They were, without doubt, of a character identical with the one described, but in an earlier stage of progress.

I adopted a simple local treatment, generous diet, with iodine and sarsaparilla, as a constitutional treatment, and hope, by this course, to avert any further destruction of tissue.

The patient has been strictly temperate since the first operation, with a single debauch, since which time the tumors spoken of have made their appearance.

CASE 3d.—Mr. James Wilson, aged 25, a stone-cutter by trade, while handling a pistol, which had been loaded for a long time, caused an explosion. The muzzle occupying the palm of the left hand, caused the contents to enter at that point, lacerating to an alarming extent the entire soft parts of the palm—literally crisping the mass of flexor tendons, and destroying both superficial and deep palmer arch.

The contents, both ball and wadding, were lodged in the soft tissues on the anterior surface of the arm, about two and a half inches above the wrist.

The hand, was so much lacerated, much of the soft parts having been entirely torn away, the interosseous spaces filled with fragments of tissue and powder, that hope of saving the hand, or any part of it, seemed to be out of the question.

The patient implored me to save it, if only enough to hold his stone chisel, as it was his only means of support.

I concluded to make the effort, hoping there might be vitality kept up by the feeble circulation of the branches of the posterior transverse carpal, a branch of the superficialis volæ, which gives off branches to supply the interosseous spaces, as well as the dorsal aspect of the fingers.

I proceeded to remove the little finger, with its metacarpal bone, which had been broken, hoping by this means to secure a better flap, one which would cover part, at least, of the remaining metacarpal bones I then ligated the arteries of the superficial and deep palmer arch—removed the ball and wadding from the fore arm, cleansed the wound as thoroughly as possible and dressed it. This done, I directed the patient (who was then, and had been, under the influence of alchoholic stimulants,) to keep his room, observe quiet, constant use of cold applications &c., &c. To my astonishment the patient, in the evening, several hours after the accident, called at my office, complaining of great pain, having been wandering around the streets nearly the entire day, without having made the necessary applications, as directed.

The hand and arm became enormously swollen, erysipelatous symptoms presented themselves, which, with difficulty, were controlled. Abcesses were found at different points, and at different times, rendering the safety of the extremity almost entirely hopeless. With all these symptoms, a persevering treatment was continued, not only in sustaining the part, but the general system.

In a few weeks it began to assume a healthy character, and in some two months the patient was restored to health, with a hand, not very useful, I acknowledge, but one better adapted to stone-cutting than even one of Palmer's best artificial hands.

I confess I had but little hope of saving this ragged specimen. The result in this, and many other cases, only goes to establish the fact that we should never resort to amputation until all hope is despaired of, not only by the surgeon in attendance, but by a counsel, which should always assist in determining the propriety of an operation.

Be not hasty in submitting a patient to the ordeal of an operation; "for he is the better surgeon who saves the limb, than he who cuts it off, no matter how skillfully the operation may be performed."

Case 4.—Mr. N. H——, aged 30, a farmer by occupation, had his hand severely lacerated by coming in contact with the teeth of a threshing machine, which he was at the time attending. The last phalanx of the thumb, with the three fingers on the index side, were entirely torn away, the little finger having been almost severed. The case was dressed, and in the care of a respectable physician in this vicinity, for three or four days, when the patient came into my charge.

I continued the treatment by following with the same dressings, but was unable to save the little finger, gangrene having taken place. The wound had assumed an unhealthy appearance, no disposition to healthy granulation, with extensive sloughing, which led me to suspect difficulty of the phalangeal extremities of the metacarpal bones.

After the removal of the little finger, which was performed by cutting through its metacarpal bone, near the extremity, I examined the entire wound and was astonished to find the soft structure of the entire flaps to have sloughed, leaving nothing but the skin covering the extremity of the bones, two of which were fractured, their uneven surfaces giving rise to this condition.

Here was another case requiring nicety of judgment in deciding as to the point of removal. A part of the hand being better than none, with a hope of saving the remaining phalanx of the thumb, I decided—assisted by the consultation of my friend, Dr. Page—to remove, at the outer third of the metacarpal bones. The sloughing had not been so extensive but that it allowed sufficient flap at that point. After the operation, the simple water dressings were applied, and nearly the entire wound healed by the first intention—giving the patient a useful and sightly extremity.

